# Preparation of an emergency-ready health workforce; a call for papers

**DOI:** 10.2471/BLT.26.296899

**Published:** 2026-08-01

**Authors:** Flavio Salio, Abdi Rahman Mahamud, Sara Hersey, Chikwe Ihekweazu

**Affiliations:** a Health Emergencies Programme, World Health Organization, Avenue Appia 20, 1211 Geneva 27, Switzerland.

Global health emergencies expose critical workforce gaps. Recent crises demonstrate that health systems often lack sufficient trained personnel and the mechanisms to rapidly mobilize them during emergencies. The *World Health Organization (WHO) pandemic agreement*^1^ represents a global commitment to strengthen pandemic prevention, preparedness and response. The agreement recognizes that strengthening health systems and workforce capacity is essential to preparedness. The agreement urges Parties to build resilient health systems, including sustaining and ensuring surge capacity for health and public health functions, and to safeguard, invest in and sustain a skilled, trained and competent health and care workforce. These provisions signal a clear global mandate to elevate workforce readiness as a pillar of health security. While the agreement and amendments to the *International health regulations*^2^ provide the overarching framework and guiding principles for international cooperation, their effectiveness depends on the development of systems and operational mechanisms at national, regional and global levels.

National governments need strong institutional capacity underpinned by the governance, legal, regulatory and financial authorities to conduct their emergency preparedness and response responsibilities. National public health agencies frequently provide the backbone for national emergency response, including a skilled workforce and the planning, management, learning and leadership structures.^3^ Global frameworks such as the WHO Global health emergency corps^4^ provide a standardized approach to guide countries in developing a public health workforce that is coordinated, cross-trained and ready to respond in the event of a crisis.

Chronic understaffing, maldistribution, burnout, regulatory rigidity and limited cross-border coordination continue to undermine efforts towards shock-responsive health systems during emergencies.^5^^,^^6^ These systemic constraints limit the ability of health systems to rapidly support and mobilize trained personnel when crises occur. Drawing on the WHO global health emergency corps framework,^7^ four interrelated dimensions illustrate the main weaknesses that must be addressed.

First, leadership. Health emergency responses are often hampered by fragmented command structures and siloed decision-making across institutions and sectors. Many countries face limited leadership capacity for complex emergency coordination, including decision-making under uncertainty, managing multisector responses and facilitating cross-border collaboration. Strengthening and institutionalizing connected leadership, empowering national public health agencies and ensuring interoperability across emergency operation centres can promote coherent decision-making, rapid coordination and seamless engagement with regional and global counterparts.

Second, surge capacities. Activation of surge mechanisms remains challenging in many settings due to weak institutional systems, including unclear activation triggers, bureaucratic delays and fragmented authority. Workforce information systems are often incomplete or poorly integrated, limiting real-time visibility of available personnel and slowing surge mobilization. Countries therefore need interoperable, predictable and high-quality surge capacities that can be rapidly activated at the national level and seamlessly integrated with regional and global surge mechanisms when required.

Third, frontline emergency workforce. National institutions often lack the required skill sets in-house to deliver on the specialized functions needed to support alert, detection, investigation and response during emergencies. Functional workforce mapping, aligned with essential emergency response functions and integrated into national workforce registries and emergency operations planning, is necessary. Doing so provides the basis for identifying capability gaps, addressing operational needs and enabling rapid deployment. In many settings, workforce capacity is further fragmented by health system privatization and limited mechanisms for effective public–private coordination during crises.

Finally, sustainability. Financing tends to be short-term and reactive, treating emergency workforce spending as a temporary expenditure rather than a long-term investment. Limited public financing and innovation capacity further constrain governments’ ability to scale and sustain emergency workforce systems. Developing a dual-use emergency workforce architecture that strengthens both surge response and routine health system functions and that is housed within national public health agencies, can improve efficiency and ensure preparedness remains functional between shocks.

Workforce challenges are particularly pronounced in fragile and conflict-affected settings, where protracted crises further impede a country’s ability to rapidly identify, mobilize and deploy personnel with the competencies and skills required for emergency response.^8^

Strengthening the health emergency workforce needs more than increasing headcount: it requires strong institutions, engaged leadership, competency-based workforce development, interoperable systems, sustainable financing and continuous exercising and sustaining operational readiness. Stronger evidence, documented operational, governance and management experience, and coordinated workforce approaches to support effective emergency response are needed. The *Bulletin of the World Health Organization *encourages submissions on an ongoing basis to all its sections to shape policy and build an emergency-ready health workforce. 
